# Relevance of BRAF Subcellular Localization and Its Interaction with *KRAS* and *KIT* Mutations in Skin Melanoma

**DOI:** 10.3390/ijms222111918

**Published:** 2021-11-03

**Authors:** Marius-Alexandru Beleaua, Ioan Jung, Cornelia Braicu, Doina Milutin, Simona Gurzu

**Affiliations:** 1 Department of Pathology, George Emil Palade University of Medicine, Pharmacy, Sciences and Technology, 38 Gheorghe Marinescu Street, 540139 Targu Mures, Romania; marius.beleaua@umfst.ro; 2 Department of Pathology, Clinical County Emergency Hospital, 540139 Targu Mures, Romania; jungjanos@studium.ro (I.J.); doina.milutin@spitalmures.ro (D.M.); 3 Research Center for Functional Genomics, Biomedicine and Translational Medicine, Iuliu Hatieganu University of Medicine and Pharmacy, 400337 Cluj-Napoca, Romania; cornelia.braicu@umfcluj.ro; 4 Research Center for Oncopathology and Translational Medicine (CCOMT), George Emil Palade University of Medicine, Pharmacy, Sciences and Technology, 540139 Targu Mures, Romania

**Keywords:** melanoma, KIT, KRAS, nuclear BRAF, gene, in silico analysis

## Abstract

Although skin melanoma (SKM) represents only one-quarter of newly diagnosed skin malignant tumors, it presents a high mortality rate. Hence, new prognostic and therapeutic tools need to be developed. This study focused on investigating the prognostic value of the subcellular expression of BRAF, KRAS, and KIT in SKM in correlation with their gene-encoding interactions. In silico analysis of the abovementioned gene interactions, along with their mRNA expression, was conducted, and the results were validated at the protein level using immunohistochemical (IHC) stains. For IHC expression, the encoded protein expressions were checked on 96 consecutive SKMs and 30 nevi. The UALCAN database showed no prognostic value for the mRNA expression level of KRAS and BRAF and demonstrated a longer survival for patients with low mRNA expression of KIT in SKMs. IHC examinations of SKMs confirmed the UALCAN data and showed that KIT expression was inversely correlated with ulceration, Breslow index, mitotic rate, and pT stage. KRAS expression was also found to be inversely correlated with ulceration and perineural invasion. When the subcellular expression of BRAF protein was recorded (nuclear vs. cytoplasmatic vs. mixed nucleus + cytoplasm), a direct correlation was emphasized between nuclear positivity and lymphovascular or perineural invasion. The independent prognostic value was demonstrated for mixed expression of the BRAF protein in SKM. BRAF cytoplasmic predominance, in association with KIT’s IHC positivity, was more frequently observed in early-stage nonulcerated SKMs, which displayed a low mitotic rate and a late death event. The present study firstly verified the possible prognostic value of BRAF subcellular localization in SKMs. A low mRNA expression or IHC cytoplasmic positivity for KIT and BRAF might be used as a positive prognostic parameter of SKM. SKM’s BRAF nuclear positivity needs to be evaluated in further studies as a possible indicator of perineural and lymphovascular invasion.

## 1. Introduction

Skin melanoma (SKM) has an incidence of approximately 1.7% of the globally diagnosed new cases of malignancies compared to non-melanoma cancers of the skin, which represent 6.2%. Despite this discrepancy, SKM has the highest mortality of all skin malignancies, with a slightly higher prevalence in males [1].

Primary and metastatic melanomas are known to be driven by several gene mutations, which modulate the tumor microenvironment [2]. The most frequently encountered mutation (52%) involves the *BRAF* (*B-Raf proto-oncogene, serine/threonine kinase*) gene, being particularly represented by the V600 amino-acid residue. It is followed by the *RAS* (*RAS-type GTPase family*) family of proto-oncogenes (*NRAS*, *KRAS*, and *HRAS*) and *NF1* (*neurofibromin 1*) [3,4]. Triple wild-type SKMs, which do not harbor *BRAF*, *RAS*, or *NF1* mutations, represent about 14.69% of all SKMs [3,4].

Another gene that plays a role in the genesis and evolution of SKM is *KIT* (*KIT proto-oncogene, receptor tyrosine kinase*). According to The Cancer Genome Atlas (TGCA) network, *KIT* mutations occur in over 1.91% of patients with SKMs [3,4]. *KIT* is known to act similarly to a proto-oncogene involved in activating downstream signaling pathways, such as the phosphatidylinositol 3-kinase/protein kinase B/mechanistic target of rapamycin (PI3K/AKT/mTOR), Src signaling, and mitogen-activated protein kinase/extracellular signal-regulated kinase (MAPK–MEK/ERK) pathways [5]. *KIT* might activate cell proliferation and inhibit apoptosis [6,7]. Because the mutation rate is not as high in SKMs, few aspects are known about the possible prognostic value of *KIT* interactions with *BRAF* and the *RAS family*.

The availability of genome-wide information in public databases offers an opportunity to assess a systematic strategy for constructing and analyzing a possible interaction map revealing novel interactions of key genes in SKM [8].

Despite the worldwide development of molecular techniques, there is still a tendency of using, in clinical practice, immunohistochemical (IHC) markers as prognostic or predictive parameters. Hence, understanding the concordance between the mutation status and IHC expression of the encoded proteins remains a challenging issue. Moreover, the importance of the subcellular expression (nuclear vs. cytoplasmatic vs. mixed-nucleus + cytoplasm) of the abovementioned markers is not known.

Proteomics expression is reported to vary between different genes depending on the RNA-to-protein ratio, which is most frequently represented by hundreds to thousands of protein copies per mRNA molecule. However, previous studies have focused on specific cell lines and their tissue, whereas skin or melanocytic cell lines were not included [9].

Although these facts lead to the necessity to further investigate skin and melanocytic cell lines, the literature suggests that the RNA-to-protein ratio should be used to predict protein expression levels for specific genes, excluding misleading ratio factors, which may influence transcriptomics. This is related to the noncoding RNAs or target-modified/miscleaved peptides, which should be filtered to avoid protein quantification biases [9]. However, because the probability of these misleading ratio factors to enhance protein copy numbers is mainly lower than 1.5-fold and, in almost all cases, below twofold if the correct quantification index is used, the prediction error is not significantly improved [10].

In this study, we used public databases to investigate the mutational pattern and mRNA expression of *BRAF*, *KRAS*, and *KIT* in SKM, along with their co-expression network. The results were validated by protein levels using IHC stains, which were performed on a representative cohort. The possible prognostic value of the subcellular expression of BRAF protein, which was firstly determined in this study, adds supplementary value to the paper.

## 2. Materials and Methods

### 2.1. In Silico Analysis of Public Databases Regarding SKMs

*BioPortal:* The cBio Cancer Genomics Portal (http://cbioportal.org) is an open-access resource for the interactive investigation of multiple genomics datasets, allowing researchers to examine and validate different hypotheses. The *BRAF*, *HRAS*, *KRAS*, *NRAS*, and *KIT* mutations in melanoma were obtained according to the cBioPortal’s online instructions. The molecular analysis performed in the 14 studies focused on SKMs, which is available on cBioPortal, refers to mutation, amplification, and deletion of the abovementioned genes [11].

#### 2.1.1. Gene Expression and Survival Analysis of BRAF, KRAS, and KIT in SKMs

We examined the correlation between gene expression profile and survival rate of patients with SKMs using the UALCAN database (http://ualcan.path.uab.edu/), which was accessed on 2 May 2021 [12]. The main inconvenience regarding the gene expression level is related to the fact that there is only one normal tissue dataset available, which makes it impossible to perform an appropriate and conclusive comparison between normal and tumor melanocytic tissue, even though an evaluation of the differences between primary tumor (*n* = 104) and metastatic case (*n* = 368) expression levels was relatively easily to conduct. This database also provided us with valuable preliminary data on mRNA level, which can be used as an additional validation step for mRNA or protein expressions.

#### 2.1.2. mRNA–miRNA Network Interaction

The mRNA–miRNA network analysis was generated using the miRNET online tool, a comprehensive tool used for multiple functional associations through network-based visual analysis [13,14,15].

### 2.2. Protein Expression Levels of BRAF, KIT, and KRAS

To validate the gene expression interaction map obtained from the UALCAN database, we performed IHC stains on representative samples of formalin-fixed paraffin-embedded (FFPE) tissues from 96 consecutive patients diagnosed with SKM. They were collected from the Tumor Archive of the Department of Pathology of the Emergency Clinical County Hospital of Targu-Mures, Romania. This retrospective study was approved by the Ethics Committee of the “George Emil Palade” University of Medicine, Pharmacy, Sciences, and Technologies of Targu-Mures, Romania.

The 96 samples were represented by surgically excised invasive-type SKMs from patients who underwent surgery between 2011 and 2018. Cases with no available follow-up information, inoperable cases, or those with positive margins, the same as cases with preoperative neoadjuvant therapy, were excluded from the database. We selected patients with at least 6 months survival length after surgery.

Consecutive cases of clinically benign melanocytic lesions, excised between 2016 and 2018 in the Departments of Adult Surgery, Plastic Surgery, and Pediatric Surgery, for aesthetic reasons, because of their proneness to repetitive traumas, or due to their suspicion for malignancy (*n* = 30), served as a control group. No synchronous or metachronous tumors were included. The benign nature of the lesions was based on the histopathological reports and, to certify the diagnosis, all cases were re-evaluated in a blinded fashion by three pathologists (S.G., I.J., M.-A.B.).

Before performing IHC stains, histological re-evaluation of SKMs was also done and all the cases were restaged based on the fourth edition of the WHO Classification of Skin Tumors [16] and eighth edition of the American Joint Committee on Cancer (AJCC) [17]. We found no underdiagnosed cases of nevi. Despite a general subjective susceptibility for misinterpretation, the dysplastic nevi presented mild dysplasia and were included into class 1 according to the Melanocytic Pathology Assessment Tool and Hierarchy for Diagnosis (MPATH-Dx) diagnostic schema [18], which presents a 92% interobserver accuracy of diagnosis correctitude in skin biopsies [19]. Our cases were represented by cutaneous sections with a low Ki67 proliferation index, thus enhancing the accuracy level, as well as offering sufficient clinical information about melanocytic lesions, as previously reported [20]. Even if, for Spitz nevi, the reported interobserver accuracy level is 40% in skin biopsies [19], in our case, full slide evaluation associated with the Ki67 index enhanced the diagnostic precision.

A representative paraffin block was selected for every case of SKM, and tissue microarray (TMA) blocks were constructed using 4 mm diameter cores per each case.

After performing 3–4 μm sections, the FFPE tissues were deparaffined and rehydrated, and IHC stains were done using the EnVisionTM FLEX system (Agilent, Santa Clara, CA, USA) and a semiautomated method. Antigen retrieval was carried out with ethylenediaminetetraacetic acid (EDTA), pH 9, using the PT Link 200 Pre-Treatment Module (Agilent). Incubation of primary antibodies was performed for 60 min, followed by incubation with Dako EnVision™ FLEX/HRP detection reagent for another 30 min at room temperature. The following antibodies were used: BRAF^V600E^ (clone RM8; dilution 1:100; BioSB, Santa Barbara, CA, USA), KIT/CD117 (polyclonal; dilution 1:500; Sigma-Aldrich, St. Louis, MO, USA), KRAS (polyclonal; dilution 1:100; BioSB), and Ki67 (clone MIB-1; dilution 1:100; Agilent, Santa Clara, CA, USA). *BRAF*-mutated papillary thyroid carcinoma served as an external positive control for BRAF, whereas interstitial cells of Cajal and *KRAS*-mutated colorectal carcinoma were used as external positive controls for KIT and KRAS, respectively. The negative control was evaluated by omitting the primary antibody. Development was done with diaminobenzidine (DAB) or magenta substrate chromogens followed by nuclear counterstaining with Mayer hematoxylin.

Evaluation of IHC expression was done in a blinded fashion by three pathologists (S.G., I.J., M.-A.B.). According to the intensity of the IHC stain and the percentage of positive tumor cells, quantification of cytoplasmic expression was based on a cutoff value of 10%. Because BRAF positivity was displayed by both the cytoplasm and nuclei of tumor cells, according to the subcellular localization of this marker and criteria of quantification previously used by our team for other IHC antibodies [21], cases were grouped into negative cases, cases with positivity in the cytoplasm only (at least 10% of the tumor cells showed cytoplasmic positivity without nuclear stain), cases with nuclear positivity (at least 10% of the cells showed nuclear positivity without cytoplasm stain), and mixed SKMs (with both nuclear and cytoplasmatic positivity in at least 25% of the tumor cells).

### 2.3. Statistical Analysis and Survival Curves

Statistical analysis was performed using GraphPad Prism 9.1.0-licensed software (GraphPad Software, San Diego, CA, USA). The Kolmogorov–Smirnov test was used to evaluate the normality of distribution between variables. Correlations and associations between IHC expression of the examined markers, overall survival rate (OS), and clinicopathological factors were checked using the nonparametric Spearman and chi-squared tests. Sensitivity and specificity were also evaluated, using the Wilson/Brown hybrid correction, for each IHC marker, comparing expression between benign melanocytic lesions and SKMs. Kaplan–Meier curves and a Mantel–Cox log-rank test were used to estimate OS. A *p*-value < 0.05 with a 95% confidence interval was considered statistically significant, using two-tailed statistical tests.

## 3. Results

### 3.1. BRAF, KRAS, and KIT Mutational Landscape in Melanoma

**Figure 1:**
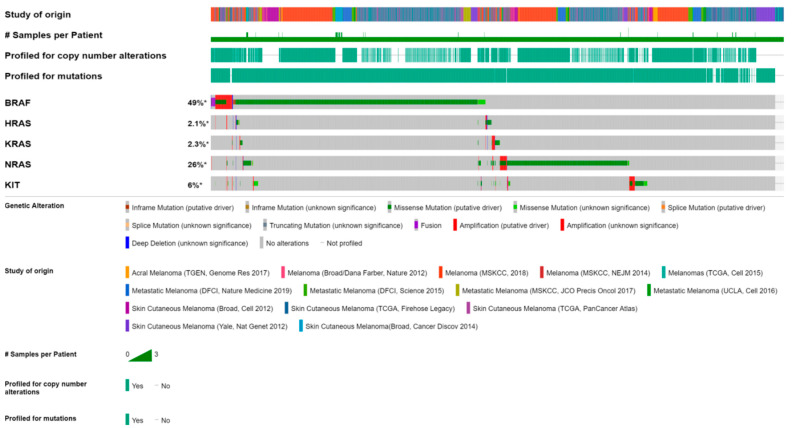
The heat map generated using cBioportal shows the mutation and copy number alteration of *BRAF*, *HRAS*, *KRAS*, *NRAS*, and *KIT* in melanoma multiple datasets. The type of the mutational status is presented in a color-coded fashion, with copy number aberration (amplification or deletion) and/or mutational state (truncating mutation, in frame mutation, missense mutation) across profiled oncogenes altered in SKMs.

The cBio Cancer Genomics Portal (cBioPortal) is one of the most comprehensive public databases and allowed us to analyze the *BRAF*, *KRAS*, and *KIT* mutation status from 14 different studies focused on melanomas. The largest number of functional mutations was observed for *BRAF* (49%) and *KIT* (6%), whereas *KRAS* mutations were found in only 2.3% of the cases (Figure 1).

### 3.2. BRAF, KRAS, and KIT mRNA Expression Level in SKMs

**Figure 2:**
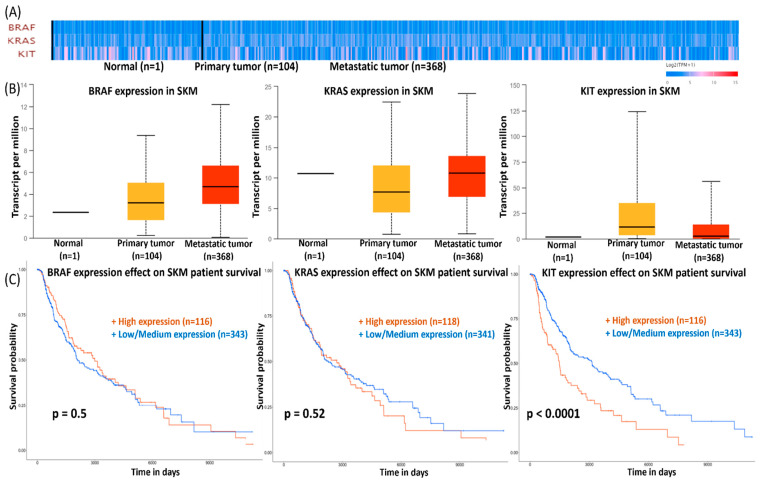
Expression level and prognostic significance of *KRAS*, *BRAF*, and *KIT* using UALCAN database: (**A**) heatmap representation; (**B**) expression level in primary and metastatic disease (TCGA samples); (**C**) survival curves comparing patients with KRAS, BRAF, and KIT high (red) and low (blue) expression in melanoma. Only KIT expression shows independent prognostic value.

**Figure A1:**
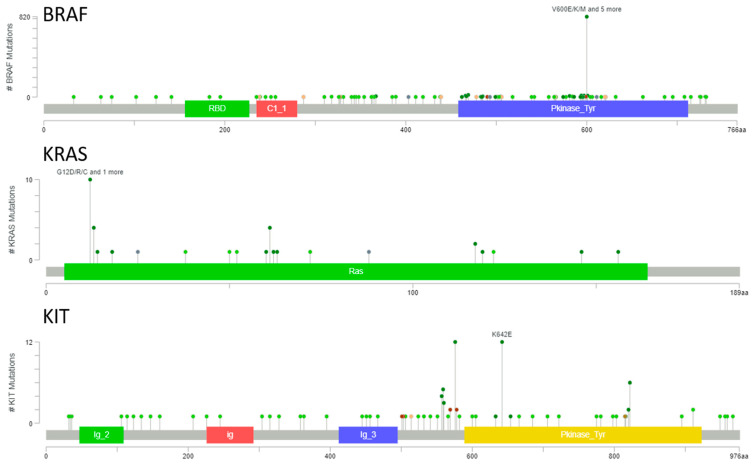
Graphical representation showing the distribution of mutations (color-coded as Figure 1) across the protein-coding regions of the three mutated oncogenes (*BRAF*, *KRAS*, and *KIT*).

**Figure A2:**
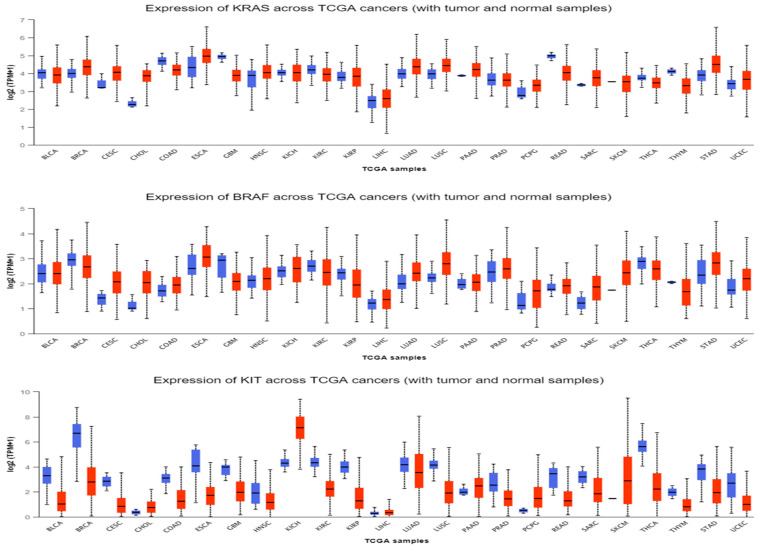
Expression levels of KRAS, BRAF, and KIT across human cancers (blue bars: normal tissue, red bars: tumor tissue).

The mRNA expression level and prognostic significance of KRAS, BRAF, and KIT were checked using the UALCAN database, in the heatmap representation (Figure 2A), normal tissue (*n* = 1), primary tumor (*n* = 104), and metastatic cases (*n* = 368). The mRNA expression of KRAS, BRAF, and KIT in various types of cancer from TCGA samples were analyzed using the UALCAN database (Figure A1 and Figure A2 in Appendix A).

BRAF and KIT expression levels of metastatic versus primary SKMs were significantly increased (Figure 2B) but they did not exert any prognostic value (Figure 2C). In contrast, KIT expression decreased in metastatic vs. primary SKMs (Figure 2B), and the KIT mRNA level, in primary tumor, was inversely correlated with OS (Figure 2C).

### 3.3. Network Interaction

**Figure 3:**
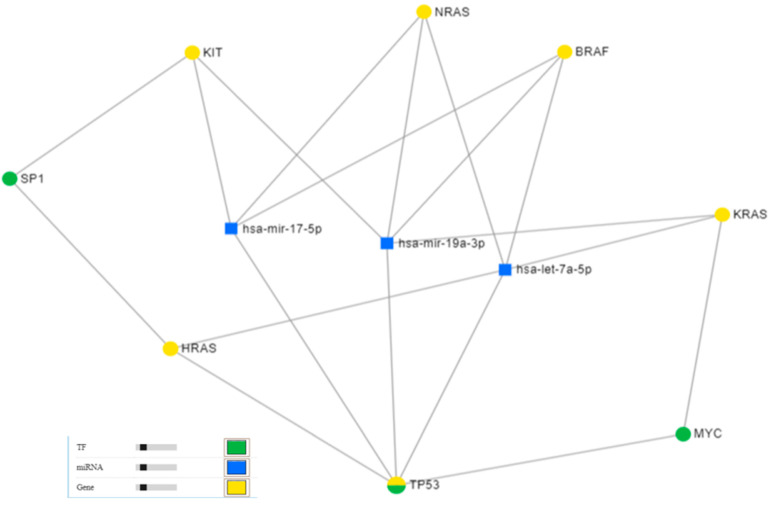
The gene–miRNA interaction in melanoma as revealed using miRNET.

The mRNA–miRNA interaction emphasizes a direct relationship between *BRAF*, *HRAS*, *KRAS*, *NRAS*, and *KIT* with the *TP53* (*tumor protein p53*) [4], as well as with two important transcription factors: SP1 (specific protein 1) and MYC (Figure 3). miRNET-targeted gene analysis showed that these genes are targeted by key miRNAs, such as miR-17-5p, miR-19a-3p, and let-7a-5p (Figure 3).

### 3.4. Protein Level Validation of the Selected Genes

In our samples, the control group was composed of six mild dysplastic and 24 benign nevi (compound—*n* = 17, junctional—*n* = 4, dermal—*n* = 3, and Spitz nevi—*n* = 7), none of them being congenital or blue nevi. They were localized on the trunk (*n* = 20), head and neck (*n* = 5), and limbs (*n* = 5) and were diagnosed in patients with a median age of 34.1 ± 8.15 years (range 1–79 years), predominantly in females (M:F = 1:2). All seven Spitz nevi were diagnosed in patients under the age of 25, ranging from 2–25 (only two cases were over 18 years old: 21 and 25 years old).

The 96 consecutive cases of SKMs affected both females and males (M:F = 1:1.08) with a median age of 63.86 ± 3 years. They were mostly localized on the trunk (*n* = 44; 45.83%), followed by the limbs (*n* = 36) and head and neck skin (*n* = 16). Nodular-type cases were predominant, showing a median Breslow index of 7.04 ± 1.7 mm (range 0.4–60 mm) and an average mitotic index of 10.31 ± 2.27 atypical mitoses/10 high-power fields. More than half of SKMs were diagnosed in stage pT4 (*n* = 50; 52.08%) (Table 1).

KIT expression was found in 6/7 Spitz, in all the mild-dysplastic nevi, and in 50% of the cases of junctional or compound nevi, whereas no positivity was encountered in dermal nevi. BRAF^V600E^ expression was found in 6/7 Spitz junctional nevi and 2/3 dermal nevi same as in half of the mild-dysplastic and junctional nevi, whereas only one out of 10 compound nevi was positive. KRAS expression was present in 9/10 compound and 3/4 junctional nevi, being expressed in all the cases of mild-dysplastic and Spitz junctional nevi; in contrast, no positivity was found in dermal nevi.

KRAS marked 83.33% of nevi and 71.88% of SKMs. KIT and BRAF were also overexpressed in nevi (63.33% and 46.66%, respectively), compared with SKMs (41.66% and 29.16%, respectively) but with no significant difference (*p* > 0.05). KRAS presented the highest sensitivity (71.88%), whereas BRAF was found to have the highest specificity (53.33%) in differentiating benign vs. malignant lesions. If, because of their rarity and higher incidence in younger people, the Spitz nevi were to be excluded from statistical assessment, the sensitivity and specificity were not significantly modified. Consecutive to Spitz nevi exclusion, KRAS sensitivity was not changed (71.88%), whereas BRAF specificity was slightly higher (65.22%). The difference in positivity percentages between non-Spitz nevi and SKMs remained nonsignificant (*p* > 0.05).

Regarding SKMs, KRAS was similarly expressed in the superficial and nodular-type SKMs, whereas it was present in almost all the lentiginous-type SKMs (7/8; 87.5%). KRAS was negatively correlated with ulceration and perineural invasion. Positive cases for KRAS were mostly nonulcerated small SKMs (≤4 mm) without neurotropism and a low number of tumor-infiltrating lymphocytes (TILs) but with a predisposition for microsatellites (Table 1).

One-third of the nodular-type SKM overexpressed BRAF (33.8%), whereas highly rare positivity was encountered in superficial or lentiginous types (16%). In contrast with KRAS-positive SKMs, cases that displayed BRAF positivity were found to be larger (>4 mm) and showed more frequent neurotropism and lymphovascular invasion (Table 1).

A positive correlation was observed between KIT positivity, histologic type, and growth phase. In contrast, KIT expression was inversely correlated with Breslow index, ulceration, mitotic rate, maximum diameter, and pT stage. The KIT-positive cases were mostly small (≤4 mm) and nonulcerated superficial or lentiginous-type SKMs diagnosed in early pT stage, with low TILs and low mitotic rate (Table 1).

There were 12 cases of SKMs (12.5%) that mutually expressed KRAS, KIT, and BRAF, which were predominantly the ulcerated nodular-type melanomas of the limbs (*n* = 10), with only two cases of lymphovascular invasion, and none showed neurotropism.

### 3.5. BRAF Subcellular Localization in SKM

**Figure 4:**
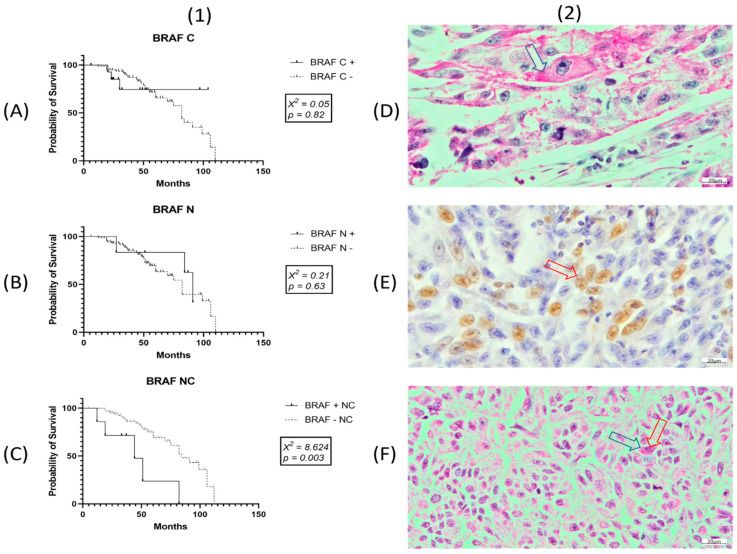
(**1**) Univariate Kaplan–Meier survival analysis shows independent prognostic value for mixed (cytoplasmic—C and nuclear—N) BRAF positivity (**C**), and not for nuclear (**B**) or cytoplasmatic (**A**) expression alone. (**2**) Representative pictures for immunohistochemistry expression of BRAF subcellular localization (20 μm): (**D**) cytoplasm only seen in red with magenta, (**E**) nuclear only marked in brown with DAB, and (**F**) mixed positivity highlighted in red with magenta (red arrow—nuclear positivity, blue arrow—cytoplasmic positivity).

BRAF was expressed in 28/96 cases of SKMs (29.16%). Most of the cases presented cytoplasmic predominance (*n* = 15; 53.57%), followed by mixed (cytoplasmic and nuclear) expression (*n* = 7) and nuclear predominance (*n* = 6) (Figure 4).

BRAF nuclear expression was positively correlated with lymphovascular (*r* = 0.28; *p* = 0.005) and perineural (*r* = 0.36; *p* = 0.0003) invasion, without correlation with any of the other examined clinicopathological factors.

Cytoplasmic predominance was directly correlated with KIT expression (*r* = 0.21; *p* = 0.03), whereas mixed positivity (cytoplasmic and nuclear) was directly correlated with death event (*r* = 0.23; *p* = 0.02), but neither correlated with any other clinicopathological factors.

### 3.6. Survival Data for SKMs

The median follow-up of the patients with SKM was 47.98 ± 5.1 months (range 6–110 months). A direct correlation was observed between death event and age (*r* = 0.38; *p* = 0.0001), Breslow index (*r* = 0.51; *p* < 0.0001), ulceration (*r* = 0.2; *p* = 0.04), mitotic rate (*r* = 0.38; *p* = 0.0001), maximum diameter (*r* = 0.49; *p* < 0.0001), and pT stage (*r* = 0.41; *p* < 0.0001).

**Figure 5:**
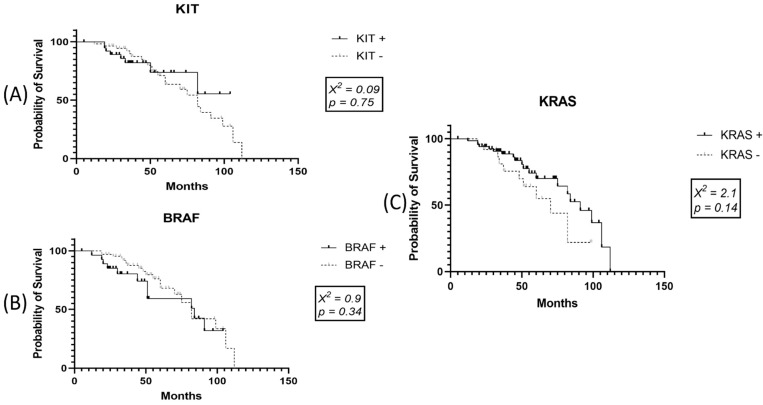
In the present cohort, univariate Kaplan–Meier survival analysis did not sustain an independent prognostic value of (**A**) KIT, (**B**) BRAF, or (**C**) KRAS expression.

Negative correlation of death event was demonstrated with histological type (*r* = −0.33; *p* = 0.001), growth phase (*r* = −0.28; *p* = 0.006), and KIT positivity. KRAS could not be used as an independent prognostic factor for OS, nor could the co-expression of BRAF/KIT, BRAF/KRAS, KRAS/KIT, or BRAF/KRAS/KIT. Although Kaplan–Meier curves did not reveal KIT as an independent prognostic factor, a correlation of death event with KIT expression showed an inverse status of the two parameters (*r* = −0.22; *p* = 0.029), suggesting that KIT positivity may exert a positive impact on OS (Figure 5).

The subcellular expression of BRAF could not be used as a prognostic factor regarding cytoplasmatic or nuclear positivity alone, whereas mixed BRAF expression highlighted this footprint as an independent prognostic factor on OS (Figure 4).

## 4. Discussion

Although the role of *BRAF*, *KRAS*, and *KIT* in transcriptomic alterations [22,23,24], tumorigenesis, and progression of several cancers has been partially elucidated [25,26,27], bioinformatics analysis in SKMs has yet to be confirmed. The present study explored the mRNA expression levels for BRAF, KRAS, and KIT, as well as their prognostic value, followed by validation through proteomic IHC expression. The obtained results might open new perspectives for the prognosis establishment of patients with SKM.

Several studies have indicated a strong correlation between protein expression and molecular analysis of *BRAF* mutations in melanomas [28]. *BRAF^V600E^* was previously reported to be considered mutated if IHC was positive. IHC cytoplasmic specificity is considered high (81–100%) and only negative or equivocal IHC reactions should be tested to exclude an existent harbored mutation [28]. To the best of our knowledge, there are no published data regarding BRAF subcellular localization (cytoplasm vs. nucleus) in SKMs. Moreover, the IHC–molecular concordance for *KRAS* or *KIT* has not been extensively studied [29].

c-KIT receptor tyrosine kinase, RAS, and BRAF are successively engaged in the downstream of MAPK pathway, which finally induces the expression of genes related to cell apoptosis, proliferation, maturation, adhesion, and motility through ERK1/2-activated transcription factors [30]. A high *KIT* mutational rate was particularly reported in mucosal melanomas [31], but differences between mucosal and SKMs were invalidated in Caucasians [32]. KIT overexpression was reported in over 50% of the cases of *KIT*-mutated melanomas but with no significant correlation between them [29]. Hence, KIT positivity is rather considered an indicator of *KIT* amplification than for an aberrant *KIT* status [33]. Evaluation of KIT expression in melanomas is also considered a possible screening method for tyrosine kinase inhibitor-targeted therapy efficacy [34].

Despite *NRAS* being the most frequently encountered mutated gene, after *BRAF*, in SKMs, it is also known that *KRAS* represents the most frequently mutated *RAS* isoform in malignancies [7,35]. Although only 2% of SKMs were reported to be associated with *KRAS* mutations, because of their possible weaker oncogenic activity in melanocytes than the other isoforms [36], several other papers highlighted the presence of *KRAS* mutations in melanoma cell lines [36,37].

Because the data are controversial due to the fact that *BRAF/KRAS/KIT* coexisting mutations were previously reported [38], particularly in primary esophageal melanomas [39], we attempted to explore the possible interaction of these three genes. Our results confirm the absence of a direct correlation among these genes but highlight a possible *BRAF/KIT* interaction if the BRAF expression is exclusively found to be cytoplasmatic, without nuclear translocation. In contrast, BRAF nuclear positivity was found to indicate a predisposition for lymphovascular and perineural invasion and was also correlated with death event. These observations emphasize the fact that the subcellular localization of BRAF can influence tumor behavior and deserves to be explored in future studies.

Online database analysis outlined a possible relationship between *BRAF*, *KRAS*, and *KIT* via their interaction with SP1 and MYC transcription factors. *SP1* works as a transcriptional activator of cell-cycle regulator genes [4,40] and influences cell apoptosis [41]. *MYC* activation can induce an intracellular network imbalance [4,42]. These interferences could represent some possible evolutive pathways of SKMs that are yet to be elucidated.

The proteomic expression of KIT, BRAF, and KRAS was previously reported in benign melanocytic lesions. KIT expression was found to be positive in all Spitz nevi, particularly in their junctional component [43], as we also report for the most part; all six junctional Spitz nevi presented KIT positivity, whereas the dermal one was negative. The literature data also show wide positivity (100%) for KIT in the dermal component of only dermal or compound nevi [43], whereas we found only 30.76% of the dermal components to be positive. KIT expression utility is enhanced by its discrepancy between benign nevi with dermal component and invasive superficial spreading melanoma [43]. The literature data reveal ubiquitarian positivity of KIT in dysplastic nevi [44], as our findings suggest.

In SKMs, BRAF^V600E^ expression was associated with a predominant dermal growth pattern, as well as with presence of intraepidermal melanocytes nesting with an increased dimension [45], similar to our finding that identified expression in most of the dermal nevi. However, we also identified BRAF^V600E^ expression in the junctional component of Spitz nevi and mild-dysplastic nevi, as well as in 50% of the junctional nevi. Only one compound nevus expressed BRAF^V600E^.

*KRAS* mutation in melanocytic nevi was previously reported in one case of congenital nevus [46], but no IHC validation was performed. Our finding offers a protein validation for a possible tumorigenic pathway through KRAS signaling [47].

In silico analysis showed a triple targeting among *BRAF*, *KIT*, and *KRAS* through miR-19a-3p, which acts as an oncogene regulating the cell cycle, as well as promoting cellular behavior through the PTEN/PI3K/AKT pathway [48]. The downregulation of miR-19a-3p is also known to enhance invasion, migration, and metastasis by activating TGF-β signaling in prostatic cancer [49] and hepatocellular carcinoma [50,51]. We did not find previous reports regarding the significance of high levels of miR-19a-3p in SKMs, but its expression was observed in the hair root of patients with psoriasis, as well as in those with both psoriasis and SKM [52]. Perhaps there is a relationship between the downregulation of miR-19a-3p and SOX10 (*SRY-box transcription factor 10*) suppression, both causing TGF-β signaling activation in different types of cancers [49,53]. Before chemotherapy, SOX10/SOX11 double positivity was reported to be directly correlated with lymphovascular invasion of SKM cells [54]. In SKMs treated with BRAF and MEK inhibitors, *SOX10* suppression was found to activate TGF-β signaling and induce resistance to oncological drugs [4,53]. These data confirm the role of miR-19a-3p in modulating BRAF, but the precise mechanism is not known.

Direct targeting of miR-17-5p was found for *BRAF* and *KIT*, and it was previously reported to act as an oncogene [55,56]. Despite its reported oncogenic activity, miR-17-5p downregulation in resistant BRAF and MEK inhibitor melanoma cell lines could act as a tumor suppressor through a lack of post-transcriptional protein death ligand 1 (PD-L1) regulation and overactivation of the Wnt-β catenin AKT/PI3K pathway [57], which is known to modulate the melanoma microenvironment [2]. PD-L1 regulation depends on the Janus kinase/signal transducer and activator of transcription (JAK/STAT) pathways controlled by MAPK pathways, and its expression is increased in BRAF and MEK inhibitor-resistant metastatic melanomas. PD-L1-positive melanomas are more aggressive, and the silencing of PD-L1 leads to miR-17-5p overexpression, which induces a less effective wound repair [57]. miR-17-5p overexpression was also previously reported to activate the TGF-β signaling pathway, increasing progression and metastasis through the Runt-related transcription factor 3 (RUNX3)/MYC/TGF-β1 positive feedback loop [58]. miR-17-5p regulates PI3K/AKT/mTOR and RAS/MAPK/ERK genes and is directly correlated with tumor stage and aggressiveness of pediatric brain tumors [59].

We found a direct targeting of let-7a-5p by *BRAF* and *KRAS*. let-7a-5p belongs to the let-7 family, which has already been reported in different types of malignancies, including melanoma, presenting a lower expression compared to normal tissues and regulating downstream genes. let-7b, a co-family member of let-7a-5p, was found to reduce cell-cycle progression and inhibition of anchorage-independent growth [60]. let-7b was significantly decreased in melanoma compared to benign melanocytic lesions and also presented a significant association with key clinicopathological factors, such as Breslow index, ulceration, and AJCC pT staging [60]. let-7a-5p expression was previously reported to be directly correlated with OS standing as an independent prognostic factor in melanoma [61] but was inversely correlated with OS in lung malignant tumors [62]. High expression was also found to prevent lung adenocarcinoma by inhibiting keratin 5 (KRT5) expression [63]. The downregulation of let-7a-5p is known to influence cancer aggressiveness. In colorectal cancer, let-7a-5p downregulation can be predictive of a worse outcome [61] by enhancing proliferation and vascular invasion [64,65]. *NEAT1* (*Nuclear paraspeckle assembly transcript 1*), a long noncoding RNA, was reported to interact with let-7a-5p, with both being inversely correlated [4]. Let-7a-5p overexpression knocked down *NEAT1*, resulting in MAPK pathway inhibition and suppressing tumor growth in nasopharyngeal carcinomas if cisplatin treatment was ongoing [66].

The limitations of this study consist of the small number of cases, lack of correlation between the obtained data and the molecular results, and the existence of only one normal skin dataset in the online database. Another limitation is represented by the heterogeneity of the control group (high incidental diagnosis of Spitz nevi) which does not reflect the incidence of the histopathological subtypes of the benign melanocytic lesions. However, this study firstly aimed to confirm the data from public databases as a starting point for further research. The innovative aspect refers to the possible prognostic role of BRAF subcellular localization in SKMs, the significance of which needs to be confirmed in further cohorts.

## 5. Conclusions

In this study, we reported, for the first time, the BRAF subcellular classification in SKMs, outlining the fact that its localization (only nuclear, only cytoplasmic, or mixed expression) might serve as a prognostic indicator of this form of skin cancer. Although BRAF nuclear positivity is an infrequent event, it might indicate aggressive behavior and perhaps an increased risk for resistance to anti-BRAF therapy.

## Figures and Tables

**Table 1:** Correlations between immunohistochemical expression of BRAF, KRAS, and KIT and clinicopathological parameters (TIL = tumor-infiltrating lymphocytes).

Parameters	*n* (%) 96	BRAF (28 +)				KRAS (69 +)				KIT (40 +)			
		+	−	*r*	*p*	+	−	*r*	*p*	+	−	*r*	*p*
Gender													
Male	46 (47.91%)	10	36	−0.16	0.12	30	16	−0.14	0.16	16	30	−0.13	0.19
Female	50 (52.09%)	18	32			39	11			24	26		
Age (years)													
≤60	34 (35.42%)	8	26	0.11	0.28	25	9	−0.13	0.19	17	17	−0.17	0.09
>60	62 (64.58%)	20	42			44	18			23	39		
Histologic type													
Nodular	71 (73.95%)	24	47	−0.17	0.09	50	21	0.03	0.75	23	48	0.34	0.0007
Superficial	17 (17.7%)	3	14			12	5			14	3		
Lentiginous	8 (8.35%)	1	7			7	1			3	5		
Breslow thickness													
≤1 mm	17 (17.7%)	4	13	0.17	0.09	15	2	−0.19	0.05	13	4	−0.21	0.03
>1 to ≤2 mm	11 (11.45%)	2	9			9	2			7	4		
>2 to ≤4 mm	14 (14.58%)	2	12			9	5			3	11		
>4 mm	54 (56.27%)	20	34			36	18			17	37		
Ulceration													
Present	71 (73.95%)	22	49	0.07	0.49	47	24	−0.2	0.04	22	49	−0.36	0.0003
Absent	25 (26.05%)	6	19			22	3			18	7		
Microsatellites													
Present	19 (19.79%)	7	12	0.09	0.33	10	9	−0.18	0.07	7	12	−0.03	0.76
Absent	77 (80.21%)	21	56			59	18			33	44		
Mitotic rate (mm^2^)													
<10	64 (66.67%)	18	46	0.15	0.12	46	18	−0.001	0.99	31	33	−0.3	0.002
≥10	32 (33.33%)	10	22			23	9			9	23		
TILs													
Present	69 (71.88%)	19	50	−0.01	0.89	46	18	−0.18	0.08	27	42	−0.003	0.002
Absent	27 (28.12%)	9	18			23	9			13	14		
Lymphovascular invasion													
Present	21 (21.88%)	10	11	0.21	0.03	17	4	0.09	0.33	6	15	−0.14	0.15
Absent	75 (78.12%)	18	57			52	23			34	41		
Neurotropism													
Present	9 (9.37%)	5	4	0.18	0.07	4	5	−0.2	0.04	2	7	−0.13	0.2
Absent	87 (90.63%)	23	64			65	22			38	49		
Tumor regression													
Present	31 (32.29%)	7	24	−0.1	0.31	24	7	0.07	0.47	15	16	0.08	0.39
Absent	65 (67.71%)	21	44			45	20			25	40		
TNM stage													
≤pT2	29 (30.21%)	6	23	0.16	0.11	7	22	−0.16	0.1	20	9	−0.3	0.002
≥pT3	67 (69.79%)	22	45			62	5			20	47		

## Data Availability

Not applicable.

## Appendix A

To reveal the transcriptional levels of BRAF, KRAS, and KIT in SKM, Gene Expression Profiling Interactive Analysis (GEPIA) was also conducted (Figure A1 and Figure A2).
